# Management of an unresponsive case of HSV keratouveitis with trabeculectomy and DSAEK: A case report

**DOI:** 10.1016/j.ijscr.2022.107505

**Published:** 2022-08-13

**Authors:** Mariya B. Doctor, Simmy Chaudhary, Sirisha Senthil, Sayan Basu

**Affiliations:** a The Cornea Institute, KAR Campus, L V Prasad Eye Institute, Hyderabad, Telangana, India; b V S T Centre for Glaucoma Care, KAR Campus, L V Prasad Eye Institute, Hyderabad, Telangana, India; c Prof. Brien Holden Eye Research Centre (BHERC), L V Prasad Eye Institute, Hyderabad, Telangana, India

**Keywords:** Herpes simplex virus, HSV trabeculitis, Endothelitis, Trabeculectomy, Endothelial keratoplasty, Case report

## Abstract

**Introduction and importance:**

This case demonstrates an unusual presentation of Herpes simplex virus (HSV) ocular infection and the challenges faced during the management of its complications.

**Presentation of the case:**

A thirty year-old lady, a steroid responder with HSV keratouveitis, was referred for non-response to treatment with the prophylactic dose of oral acyclovir and acetazolamide. She presented with large epithelial bullae, anterior chamber reaction, and raised intraocular pressure in her right eye. Initially, she responded to the therapeutic dose of oral acyclovir, but on follow-up visits, she developed high intraocular pressures of up to 45 mmHg on maximum medical therapy. Hence, trabeculectomy with mitomycin-C was performed. One year later, she developed corneal endothelial decompensation, for which a Descemet's stripping automated endothelial keratoplasty (DSAEK) was done. Eight months post-operatively, she had a best corrected visual acuity of 20/20, clear corneal graft, quiet anterior chamber, and well-controlled intraocular pressures.

**Discussion:**

HSV trabeculitis is associated with inflammation of the anterior chamber, endothelitis and raised intra-ocular pressure. A combination of anti-viral, anti-inflammatory, and anti-glaucoma medications helps in the management. However, glaucoma filtration surgery is often needed to the control intra-ocular pressure. Chronic recurrent episodes eventually lead to endothelial failure and demand endothelial keratoplasty (EK). It is prudent to adopt certain measures to perform EK in these phakic eyes without causing any iatrogenic damage to the filtration bleb as well as to the clear crystalline lens.

**Conclusion:**

This case highlights the difficulties of treating HSV-related keratouveitis with uncontrolled glaucoma, problems of associated steroid response, and complexities in performing corneal endothelial procedures in young phakic patients especially post-trabeculectomy.

## Highlights

• HSV infecting the uveal tract is likely to infect the endothelium and trabecula.
• HSV trabeculitis is associated with endothelitis and high intraocular pressure, which is not controlled with maximum medical management.
• Glaucoma filtration surgery helps in controlling intraocular pressure.
• Chronic cases develop endothelial dysfunction and demand endothelial keratoplasty.
• Endothelial keratoplasty in a phakic eye needs a strategical approach intra-operatively.

## 1 Introduction

Viral anterior uveitis (VAU) is characterized by anterior segment inflammation, elevated intraocular pressure (IOP), keratitis, and patches of iris atrophy. The main viruses causing VAU are Herpes simplex type 1 (HSV-1), Varicella-Zoster (VZV), and Cytomegalovirus (CMV). Herpetic AU is the most common, seen in about 5–10 % of all uveitis cases in the western world and 0.9–8.3 % of all infectious uveitis in India [1], HSV AU is usually a recurrent disease and leads to intermittent raised intraocular pressures. Treatment involves antiviral, anti-inflammatory and anti-glaucoma medications. In a few unresponsive cases, or in steroid responders, the treatment is complicated since the intraocular pressure remains uncontrolled with pharmacological therapy and requires surgical intervention in the form of filtrating surgeries. Also, the virus can cause corneal endothelial decompensation which could require corneal transplantation [2]. We highlight the difficulties faced in the treatment of one such unresponsive case, and the challenges posed while performing corneal transplantation in these patients. This case report is as per the SCARE-2020 criteria [3].

## 2 Presentation of case

**Fig. 1:**
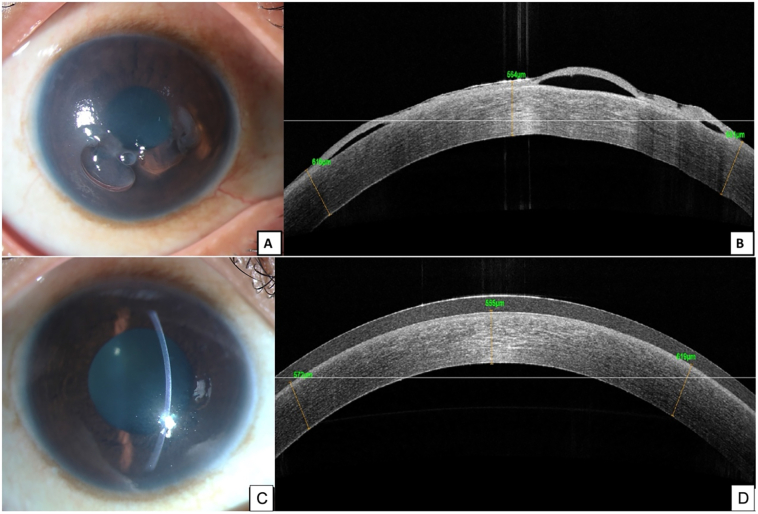
(A and B). Image of the right eye at the time of presentation. A. Diffuse slit lamp image of the right eye showing multiple large paracentral epithelial bullae with. B. Anterior segment OCT image showing corneal epithelial bullae along with corneal edema. (C and D) Image of the right eye after 3 days of starting therapeutic dose of oral antiviral therapy. C. Diffuse slit lamp image of the right eye with complete resolution of epithelial bullae, compact stroma and BCL in situ. D. Anterior chamber OCT images showing the compact cornea with no corneal edema or bullae.

**Fig. 2:**
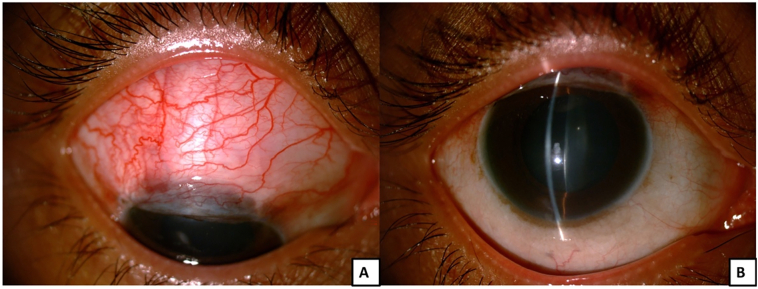
Image of right eye post trabeculectomy. A. Diffuse slit lamp photo showing an elevated, diffuse conjunctival bleb in the superior bulbar conjunctiva. B. Slit-view image of the cornea of the same eye on day one post trabeculectomy. Minimal edema is noticed in the central cornea, while rest of the cornea appears clear.

**Fig. 3:**
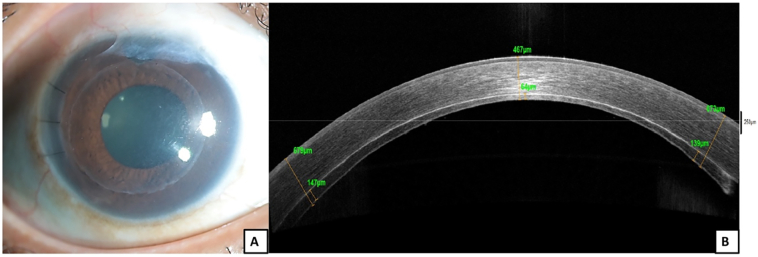
Image of right eye post endothelial keratoplasty. A. Diffuse slit lamp image with clear cornea, attached DSAEK lenticule, compact stroma, three interrupted sutures at the main section and superior low-lying, diffuse conjunctival bleb. B. Anterior segment OCT showing a well attached DSAEK lenticule with compact corneal stroma.

**Fig. 4:**
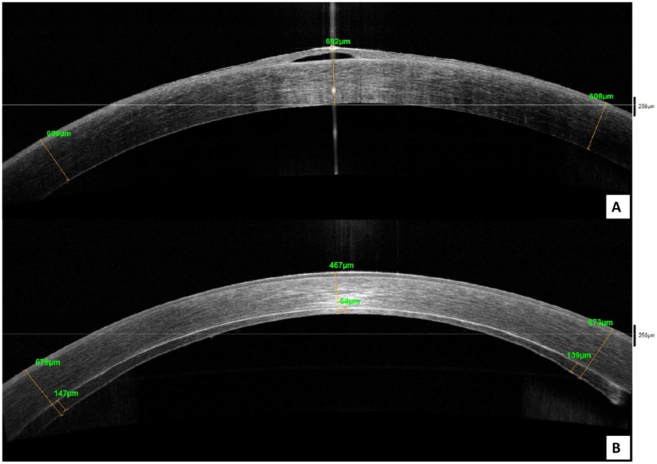
Anterior segment OCT of the right eye before and after DSAEK surgery. A. Showing the corneal edema and epithelial bullae before undergoing DSAEK surgery. B. Showing the compact corneal stroma, and well attached DSAEK lenticule post-operatively.

A thirty year-old lady presented to us with complaints of pain and watering from her right eye for the past one month. There was no history of ocular trauma. She had multiple similar episodes in the past year and a half in the same eye for which ophthalmic consultation was taken locally, where she was diagnosed with recurrent viral trabeculitis. An anterior chamber tap had revealed PCR test positivity for HSV-1. She was referred to us in view of her poor response to intraocular pressure (IOP) control with medical treatment which was further complicated by steroid responsiveness. She was on oral acyclovir 400 mg tablets twice a day, along with topical brimonidine tartrate 0.2 % and timolol maleate 0.5 % eye drops since the past 1 year. There was no significant family history. On presentation, her best corrected visual acuity (BCVA) in the right eye (RE) was 20/600 and N36, and she was 20/20 and N6 in her left eye (LE). Slit lamp biomicroscopic examination of the RE revealed large corneal epithelial bullae in the background of diffuse stromal edema, few keratic precipitates (KPs), mild anterior chamber reaction, and intraocular pressures (IOP) of 20 mmHg, and normal anterior segment findings with IOP of 14 mmHg in the left eye (Fig. 1A and B). We placed a large diameter bandage contact lens and started the patient on a therapeutic dose of oral acyclovir 400 mg (5 times/day) and topical and oral antiglaucoma medications (oral acetazolamide 250 mg thrice a day, topical brimonidine tartrate 0.2 % and timolol maleate 0.5 % twice daily). We also started her on topical cycloplegic (homatropine hydrobromide 5%) eye drops thrice daily. She was doing well for the initial few weeks (Fig. 1C and D), and the RE corneal condition improved in the subsequent visits, however, the IOP was uncontrolled even on maximum medications (upto 45 mmHg) at the one-month follow-up visit. Gonioscopic examination revealed open angles in all quadrants in both eyes with the right eye having grade 3–4 pigmented trabecular meshwork 360 degrees suggestive of secondary open-angle glaucoma (pigmentary glaucoma) with steroid response. When despite treatment with oral acyclovir 800 mg twice a day, oral acetazolamide 250 mg, topical brimonidine tartrate 0.2 %, timolol maleate 0.5 %, and dorzolamide 2 % twice daily, her IOP remained uncontrolled on maximum medical therapy, we decided to perform trabeculectomy with mitomycin-C in her right eye. Post-operatively she was doing well with visual acuity of 20/50, well-formed diffuse bleb, and IOP of 8 mmHg (Fig. 2A and B). However, at the one-year follow-up visit, her BCVA in the right eye had decreased to 20/100p. Her ocular examination revealed diffuse corneal stromal edema with epithelial bullae in the RE; suggestive of endothelial decompensation, hence she was planned for Descemet's stripping automated endothelial keratoplasty (DSAEK). Following DSAEK, she was prescribed oral acyclovir 400 mg five times daily for two weeks followed by maintenance of twice daily dose, and weekly tapering dose of topical prednisolone acetate 1 % from six times per day to maintenance dose of once daily for 6 months, with a combination of brimonidine tartrate 0.2 %, timolol maleate 0.5 % twice a day. Both the surgeries were performed under local anesthesia by experienced surgeons. At the final follow-up, eight months post-operatively, the DSAEK lenticule was well attached with BCVA of 20/20 (Figs. 3A, B and 4A, B) in the RE. There was no evidence of recurrence of infection, and her intraocular pressures were under control.

## 3 Discussion

Ocular HSV infection involving the uveal tract usually affects the corneal endothelium. It is suggested that the virus enters the trabeculum which is innervated by the trigeminal nerve, and subsequently involves the corneal stroma and endothelium, which are in direct continuation with the trabecula in the periphery [4], [5]. HSV trabeculitis is characterized by anterior segment inflammation associated with focal patches of iris atrophy, localized endothelitis with the corresponding deposition of fine KPs, along with raised intra-ocular pressures [6]. Corneal epithelial blebs, along with the presence of dark areas and loss of endothelial mosaic pattern are noted both on slit-lamp examination and specular microscopy [7]. This occurs due to endothelial cell edema and inflammation in response to the HSV invasion, leading to the separation of the Descemet's membrane. Histopathological findings have suggested that there occurs swelling and vacuolization of cytoplasm of the corneal endothelial cells along with loss of the normally compact arrangement which eventually leads to large empty patches due to loss of these necrotic cells without replacement [8]. Also, the corneal endothelial cell density is usually lower than the fellow eyes in chronic recurrent cases [8].

The prevalence of raised IOP in these cases is noted to be very high, varying from 50 to 90 % [9]. This raised IOP usually correlates with the peak of anterior chamber reaction [8]. IOP elevation occurs due to direct involvement of the trabeculum by the virus, and inflammatory cell and debris infiltration of the trabecular meshwork leading to disruption of the lamellar arrangement and causing resistance to the aqueous outflow. In chronic cases, there occurs formation of retrocorneal membrane, and anterior synechiae which further hamper the already compromised outflow. These patients also have increased rates of steroid response [8], [9], [10].

Treatment of cases of HSV AU involves long term antiviral, anti-inflammatory and anti-glaucoma therapy. Oral acyclovir (for longer than 6 months) is said to reduce the recurrence of uveitis in these patients. After antiviral coverage is started, anti-inflammatory treatment can provide a rapid control of anterior chamber inflammation along with controlling the raised intra-ocular pressure. In patients having recurrent disease, IOP may not be controlled with maximal medical treatment (approximately 25–30 %) and these patients further require surgical intervention in the form of filtration surgery like trabeculectomy or glaucoma drainage device procedures, which provides a good IOP control [9].

Further, a recalcitrant disease can lead to corneal decompensation which would require corneal transplantation. A penetrating keratoplasty (PKP) or a lamellar keratoplasty are the options to be considered. Lamellar keratoplasty has replaced PKP for HSV endothelial disease due to lower rates of HSV recurrences, lower risks of graft rejection, better visual outcomes, and quicker visual rehabilitation [2]. A PKP procedure in a post trabeculectomy eye is difficult due to the chances of perforation of the bleb during trephination or suturing of the corneal graft. Hence an endothelial transplantation procedure must be preferred. A dilemma to decide on endothelial transplantation occurs in young patients who have clear crystalline lenses since these eyes have a high risk of cataract development and progression [11], [12]. This occurs due to a shallow anterior chamber and chances of iatrogenic lenticular trauma during insertion and unfolding of the DSAEK graft [13], [14]. Hence surgeons usually prefer a combined procedure of endothelial transplantation with clear lens extraction. However, the cataract progression can be prevented by following certain simple maneuvers such as administration of pre-operative mannitol, use of intracameral pilocarpine to constrict the pupil thereby protecting the crystalline lens, and the use of cohesive viscoelastic devices during descemetorhexis to maintain the depth of anterior chamber during the surgery [13]. We follow these precautionary steps in our cases of phakic endothelial transplants which help prevent iatrogenic trauma. However, any transplant performed in a case of HSV keratitis or keratouveitis requires long-term prophylaxis of antivirals, and yet some cases have recurrence of HSV infection. Our patient had a rare presentation of large epithelial bullae and a recalcitrant form of uveitic glaucoma which made the management difficult. Hence such cases are complex and require a long and well-planned treatment algorithm.

## 4 Conclusion

This case highlights the difficulties of treating HSV keratouveitis with uncontrolled glaucoma, problems of associated steroid response, and complexities in performing corneal endothelial procedures, especially in young phakic patients who have undergone trabeculectomy.

## Funding

Hyderabad Eye Research Foundation, Hyderabad, Telangana, India.

## Ethical approval

Ethics committee approval was not required for this manuscript because it is a clinical case report.

## Consent

Written informed consent was obtained from the patient for publication of this case report and accompanying images. A copy of the written consent is available for review by the Editor-in-Chief of this journal on request.

## Author contribution

Study concept or design: SB

Writing and revising the paper: MD, SC, SS, SB

## Registration of research studies

Not applicable.

## Guarantor

Sayan Basu.

## Declaration of competing interest

The authors have no conflicts of interest to disclose.
